# 3D Chitosan-Gallic Acid Complexes: Assessment of the Chemical and Biological Properties

**DOI:** 10.3390/gels8020124

**Published:** 2022-02-15

**Authors:** Maria Marzano, Nicola Borbone, Felice Amato, Giorgia Oliviero, Pierpaolo Fucile, Teresa Russo, Filomena Sannino

**Affiliations:** 1 Institute of Applied Sciences and Intelligent Systems Unit of Naples, National Research Council, Via Pietro Castellino 111, 80131 Naples, Italy; maria.marzano@na.isasi.cnr.it; 2 Department of Pharmacy, University of Naples Federico II, Via Domenico Montesano 49, 80131 Naples, Italy; nicola.borbone@unina.it; 3 ISBE-IT, University of Naples Federico II, 80138 Naples, Italy; giorgia.oliviero@unina.it; 4 Department of Molecular Medicine and Medical Biotechnologies, University of Naples Federico II, Via Sergio Pansini 5, 80131 Naples, Italy; felice.amato@unina.it; 5 CEINGE-Biotecnologie Avanzate, Via Gaetano Salvatore 486, 80131 Naples, Italy; 6 Department of Advanced Biomedical Sciences, University of Naples Federico II, Via Sergio Pansini 5, 80131 Naples, Italy; pierpaolo.fucile@unina.it; 7 Institute of Polymers, Composites and Biomaterials, National Research Council, Viale J. F. Kennedy 54, Pad. 20 Mostra d’Oltremare, 80125 Naples, Italy; teresa.russo@cnr.it; 8 Department of Agricultural Sciences, University of Naples Federico II, Via Università 100, 80055 Naples, Italy

**Keywords:** 3D chitosan, gallic acid, 3D chitosan-gallic acid complexes, adsorption process, spectroscopic analyses, bioactivity

## Abstract

Three-dimensional chitosan-gallic acid complexes were proposed and prepared for the first time by a simple adsorption process of gallic acid (GA) on three-dimensional chitosan structures (3D chitosan). Highly porous 3D devices facilitate a high GA load, up to 2015 mmol/kg at pH 4.0. The preservation of the redox state of GA released from 3D chitosan was confirmed by spectroscopic analyses. The antioxidant activity of 3D chitosan-GA complexes was assessed using the DPPH radical scavenging assay and was found to be dramatically higher than that of free chitosan. The mechanical property of 3D chitosan–GA complexes was also evaluated using a compression test. Finally, 3D chitosan–GA complexes showed a significant antimicrobial capacity against *E. coli* and *S. aureus*, selected, respectively, as a model strain for Gram-negative and Gram-positive bacteria. Our study demonstrated a new, simple, and eco-friendly approach to prepare functional chitosan-based complexes for nutraceutical, cosmeceutical, and pharmaceutical applications.

## 1. Introduction

The encapsulation of bioactive molecules into biopolymers aims at improving the functional properties of the former via the protective effect of the latter against light, heat, oxygen, and pH, as well as the more efficient transport of bioactives at their target destination. At the same time, the incorporation of actives into biopolymers provides the latter with valuable properties. The incorporation of plants’ polyphenols in food packaging and in cosmetic formulations are good examples of this important topic. 

Natural phenolic compounds possess significant antioxidant and antimicrobial characteristics. However, most of them are susceptible to degradation processes, are poorly soluble, and have limited bioavailability [1]. So, the incorporation of plants’ polyphenols into biodegradable biopolymers, on one hand, allows for an improvement of their stability, solubility, and bioavailability [1,2] and on the other, provides the biopolymer with characteristics useful in pharmacological [3], nutraceutical [4,5] and cosmeceutical applications [6,7]. Among the available biodegradable polymers, chitosan deserves particular attention due to the sustained release of highly bioavailable phytochemicals.

Chitosan is a cationic polysaccharide produced by the deacetylation of chitin, a polymer found in the exoskeleton of crustaceans and insects [8]. Its excellent properties such as biocompatibility, biodegradability, antimicrobial activity, and non-toxicity make chitosan an auspicious and eco-friendly material for different purposes in many fields such as agriculture, medicine, biotechnology, and the food industry [9]. Chitosan has been approved by the United States Food and Drug Administration (USFDA) as a “Generally Recognized as Safe” (GRAS) food additive [10]. It has been the first basic substance approved by the European Union for plant protection (2014/563) for both organic agriculture and integrated pest management. Moreover, chitosan-based materials can be designed in different forms, including gels, films, and porous scaffolds capable of trapping essential oils and bioactive compounds [11] thereby allowing for their use as food coatings and controlled drug-release systems [12]. 

Several design strategies to develop complex formulations and devices based on chitosan and polyanionic polymers (e.g., films, scaffolds, fibers) have been proposed [13,14,15]. In this scenario, previous studies demonstrated how chitosan and its complexes may be readily employed to develop three-dimensional (3D) structures through electrospinning and freeze-drying [15,16,17,18]. Recently, 3D printing/fused deposition modeling has also been proposed as an innovative method to obtain 3D chitosan-based structures in environmental wastewater treatment, for example as a TiO_2_ support for the photocatalytic degradation of amoxicillin in water [19]. 

Chitosan structures are generally prepared using freeze-drying technology. The structural and chemical–physical properties of 3D chitosan structures, their mechanical strength, and mass transport properties (i.e., permeability, diffusion) may be properly modulated by varying the chitosan concentration, freezing rate, molecular weight, and percentage of deacetylation [15,20].

Briefly, 3D porous structures with controlled morphological features present the possibility to design advanced devices with tailored porosity, and mechanical and mass-transport properties [19]. The internal architecture and the size and distribution of pores influence the developed devices’ characteristics, allowing them to meet the design and functional requirements.

The development of 3D porous devices offers the possibility to better satisfy several functional requirements if compared to 2D substrates [20,21]. In general, the 3D architecture provides a distribution of the binding sites and pores size in various locations within the structure, rather than only on the single substrate plane as in the 2D architecture. 

Thus, a highly porous 3D structure is desirable, and the open pores enable the flowing of liquids and the transport of molecules through the porous matrix. The major advantage of this material is its ability to accommodate active molecules of various sizes and in large quantities. Chen et al. [21] highlighted how parameters such as area-to-volume ratio, or specific surface, pore volume and dimensions could be responsible for improving adsorption capacity of 3D porous polymeric adsorbents, also affecting release mechanisms. 

In the agri-food sector, food-packaging development based on chitosan structures represents an environmentally friendly and sustainable alternative because chitosan is renewable and biodegradable [1,2]. As food packaging fulfills essential functions, such as preventing physical, chemical, and microbiological contamination and food adulteration, the food industry is continuously looking for eco-friendly and sustainable solutions. However, the unique chitosan’s macromolecular structure and its hydrogen atom donor deficiency resulted in insufficient biological activities, significantly limiting its application. For this reason, many molecular modifications such as alkylation, acylation, and grafting have been proposed as effective methods to enhance the biological activity of chitosan. In recent years, the use of chitosan functionalized with different antioxidants has been assessed. 

Composite hydrogels based on chitosan and loaded with polyphenols have been widely investigated and recently reviewed by Micale et al. [22]. Just as an example, Lišková et al. [23] developed chitosan-based injectable hydrogels enriched with phloroglucinol (PG) and gallic acid (GA) intended for applications related to bone regeneration.

Lozano-Navarro et al. [24] described the preparation of chitosan/starch films associated with natural antioxidants, proving that these materials possess promising properties for packaging in industry food. In this context, the controlled release of the film-entrapped antioxidant from the packaging to the food surface leads to a reduction in the amount of chemical preservatives required in food.

Furthermore, da Rosa et al. [25] reported the microencapsulation of gallic acid (GA) in chitosan matrices via lyophilization, thus achieving an enhancement in the antioxidant activity of chitosan. On the other hand, Jiang et al. [26] developed and studied chitin hydrogel loaded with GA for wound healing application and/or cancer treatment. Chitosan was also conjugated with GA using a crosslinking agent in the form of water-soluble nanoparticles for drug delivery applications, providing interesting information in release kinetics for the attainment of a desired drug concentration [27]. Anyway, methods such as graft copolymerization have been widely investigated to confer desirable properties onto chitosan [9]. Three types of grafting approaches, including free radical initiation, carbodiimide coupling, and enzyme catalysis are generally adopted to synthesize phenolic grafted chitosan [28]. Due to the diversity of coupled active molecular structures, their corresponding coupling methods are also different [29]. However, different coupling methods are connected to diverse coupled active molecular structures. Just as an example, Curcio et al. [30] proposed a covalent insertion of antioxidant molecules on chitosan via a free-radical grafting procedure, adopting H_2_O_2_/ascorbic acid redox pair, in an environmentally friendly and safe way. Green and economical radical initiation presents the main drawback in its low derivatization degree. Carbodiimide coupling requires functional group protection for an efficient synthesis of chitosan conjugates [31].

Chitosan conjugates that have better properties should be obtained via enzyme-assisted coupling reactions, sometimes requiring harsh reaction conditions. The oxidation of the hydroxyl group in phenolic acid could also reduce the activity of the synthetic product [29,32] 

Taking into account the previously mentioned limitations, with the aim of enhancing the functional properties and biological activity of 3D chitosan structures by incorporating 3,4,5-trihydroxybenzoic acid (the IUPAC name of GA) without the addition of any chemical agent, this work aims to obtain 3D chitosan–GA complexes, thus fulfilling the green chemistry principles. 

GA is a natural phenolic compound present in many plants, especially grapes and tea, provided with antioxidant, antimicrobial, antimutagenic, anti-inflammatory, and anticancer properties [28,33,34]. 

The aim of this study was to evaluate (i) the ability of 3D chitosan structures to complex GA via simple, noncovalent adsorption; (ii) the antioxidant properties of the complexes and the role played by the pH; (iii) the effect of drying time on the GA release kinetics; and (iv) the antimicrobial properties of 3D chitosan-GA complexes. 

## 2. Materials and Methods

### 2.1. Materials

Chitosan (CAS 9012-76-4) with a molecular weight of ~300 kDa and deacetylation degree of 85% was supplied by Heppe Medical Chitosan GmbH (Halle, Germany). Hydrochloric acid (37% ISO for analysis), sodium hydroxide and 2,2’-diphenyl-1-picrylhydrazyl (DPPH) were purchased from Carlo Erba (Milano, Italy), AppliChem (Darmstatd, Germany), and Merck KGaA (Darmstadt, Germany), respectively. Dibasic sodium phosphate (Na_2_HPO_4_), acetic acid (99.8%, Romil Pure Chemistry), 99% D_2_O for NMR, and FT-IR grade KBr were provided by Merck KGaA. Gallic acid (3,4,5-trihydroxybenzoic acid, 98.0% purity), employed as a bioactive compound, was supplied by Merck KGaA.

### 2.2. Preparation of 3D Chitosan Structures

Three-dimensional chitosan porous structures were obtained adapting a protocol described by Yang et al. [15]. Briefly, a 3% (w/v) solution of chitosan in 1% (v/v) acetic acid was prepared at room temperature and stirred overnight (4000 rpm). 

An adequate volume (10–30 µL) of the dibasic sodium phosphate (Na_2_HPO_4_) solution at 100 mg/mL concentration was added dropwise into the chitosan solution under magnetic stirring until the mixture reached a pH of 7.0–7.2.

Finally, the auto-gelling solutions were transferred into a 48-well plate and incubated at 37 °C overnight to obtain the chitosan hydrogel (3D chitosan). The obtained chitosan hydrogel was stored in a refrigerator at –80 °C for 24 h and lyophilized in a freeze dryer for 24 h.

The porosity of 3D chitosan structures was evaluated according to the method described by Kuo et al. [35] using 3D structures 10 mm in diameter and 5 mm in height. The porosity (P (%)) was defined as:

$$P\left(\%\right)=\frac{V_{p}}{V_{s}}=\frac{\left(W_{w}-W_{d}\right)/\rho_{eth}}{V_{s}}=\frac{W_{w}-W_{d}}{W_{w}-W_{0}}\times 100$$

where *V_p_*, *V_s_*, *ρ*_*eth*_, *W_w_*, *W_d_* and *W*_0_ represent the pore volume, the apparent volume of the 3D structures (total volume of pores and solid matrix), the density of ethanol (Sigma Aldrich), the wet weight of the 3D structures after immersion in ethanol, the dry weight of the structures and the weight of the structures in ethanol, respectively, and W_0_ is assumed the dry weight of the 3D structure, whilst (*W_w_* − *W*_0_) is equal to *V_s_* × *ρ*_*eth*_. 

### 2.3. Preparation of Gallic Acid Solution

GA (40 mg) was dissolved in 10 mL of ultrapure water for 2 h at room temperature under magnetic stirring to obtain a 23.5 mM solution. It was stored at 4 °C in the dark to avoid oxidation reactions. The stock solution was suitably diluted with ultrapure water to obtain following concentrations: 15.0, 7.5, 5.0, 1.0, 0.8, 0.5, 0.25, 0.15, and 0.05 mM. The analytical determination of GA was carried out by UV-vis spectrophotometer (ThermoScientific Varioskan Flash, Vantaa, Finland) at 265 nm. 

### 2.4. Preparation of 3D Chitosan-GA Complexes 

Preliminary tests were performed to determine the optimal pH at which the sorption of GA on 3D chitosan occurs. The 3D chitosan samples (100 mg) were incubated with 10 mL of 1.0 mM GA solution (low loading procedure, LL) in Falcon tubes, kept at room temperature in an Asal 708 stirrer (Asal Srl, Cernusco sul Naviglio, Italy) for 24 h in the dark. The experiments were carried out as a function of pH (3.0, 4.0, 5.0, 6.0, 7.0, 8.0, and 9.0), which was adjusted by the dropwise addition of 0.1 M HCl or NaOH solutions. At the end of the incubation period, each sample was centrifuged at 4000 rpm for 30 min, the supernatant was removed, and the precipitate was washed twice with 10 mL of ultrapure water to ensure the removal of the GA that was not adsorbed, but instead simply deposited on the surface of 3D chitosan [36]. The GA concentration in the supernatant and washings was then determined spectrophotometrically as reported in Section 2.3. The amount of GA adsorbed on 3D chitosan structures was calculated as the difference between the initially added and non-adsorbed concentration. Blanks of GA in ultrapure water at the same pH of the samples were analyzed to evaluate GA stability and adsorption on Falcon tubes. The 3D chitosan–GA complexes used for mechanical characterization, DPPH scavenging assay, release studies, and antimicrobial activity were then obtained by for the interaction of 24 h dried 3D chitosan samples (100 mg) with either 10 mL of 15.0 mM (medium loading procedure, ML) or 23.5 mM (high loading procedure, HL) GA solutions for which the pH was adjusted to 4.0 (the optimal loading pH) as reported above (Table 1). All 3D chitosan-GA samples were dried for at least 24 h before further analysis.

### 2.5. Mechanical Properties of 3D Chitosan-GA Complexes

Compression tests were performed at 25 °C in a dry state on 3D chitosan and HL-3D chitosan-GA specimens. Each cylindrically shaped specimen was characterized by a diameter (d) of 8.0 mm, and a height (h0) of 4.0 mm. All the tests were carried out on the specimens at a rate of 1 mm min^−1^ using an INSTRON 5566 testing system. The ‘apparent’ stress σ was evaluated as the force F measured by the load cell divided by the total area of the apparent cross section of the 3D structure (A = πd^2^/4), while strain ε was defined as the ratio between the height variation (Δh) of the specimen and its initial height (h0).

### 2.6. Nuclear Magnetic Resonance (NMR) Analysis

Water-suppressed ^1^H NMR spectra were recorded on a Bruker AvanceNeo 700 MHz spectrometer (Bruker, Billerica, MA, USA) equipped with a CHN triple resonance cryoprobe. 3D chitosan, GA and HL-3D chitosan-GA complex were dissolved in H_2_O/D_2_O 9:1 and the pH was adjusted to 3.5 (and to pH 6.0 for GA) by dropwise addition of 0.1 M HCl or NaOH solution at final concentration of 3.2, 15 and 3.3 mM, respectively. The spectra were acquired at 25 °C and processed using the MestReNova software package (rel. 14.2.0, Mestrelab Research, Santiago de Compostela, Spain). Proton chemical shifts were referenced to the residual water signal, resonating at 4.78 ppm. Water suppression was achieved using the excitation sculpting with the gradient routine included in the “zgesgp” pulse sequence [37]. 

### 2.7. Fourier Transform Infrared Spectroscopy (FT-IR)

Fourier transform infrared spectroscopy (FT-IR) spectra were recorded using a Jasco FT–IR 430 spectrophotometer (Jasco Europe, Cremella, Italy). The 3D chitosan, GA, and ML-3D chitosan-GA complexes were dried and ground into a powder form before the FT-IR analyses. Sample powders were mixed in a 1:100 weight ratio with KBr and pressed into a disk under vacuum. The samples were scanned from 400 cm^−1^ to 4000 cm^−1^.

### 2.8. Release of GA from 3D Chitosan-GA Complexes

The HL-3D chitosan-GA samples, obtained as described in Section 2.4, were suspended in Falcon tubes with 10 mL ultrapure water at pH 4.0, 5.0, 6.0, and 7.0 to investigate the release behavior of GA at different pH. After 24 h, the supernatants were removed, and the released GA concentration was determined by UV analysis at 265 nm. With the aim of evaluating the effect of different drying times on the release of GA from 3D chitosan-GA complexes, samples of HL-3D chitosan-GA were vacuum dried for 24 h (CH1), seven days (CH2), and 14 days (CH3). At the end of drying, the samples were transferred into Falcon tubes and mixed with 10 mL of ultrapure water at pH 6.0 (the optimal release pH). The supernatants were taken out at different time points (40, 90, 120 and 1440 min), the GA concentration was determined by UV analysis, and the GA release was quantified as follows:

$$Released\;GA\;\left(\%\right)\frac{GA_{t}}{GA_{o}}\times 100$$

where *GA_t_* is the cumulative amount of *GA* released at any time point and *GA_o_* is the initial amount of *GA* adsorbed onto the 3D chitosan pores.

### 2.9. In Vitro Antioxidant Activity by DPPH Radical Scavenging Assay

The DPPH radical-scavenging activity was assayed according to the method of Hu and coworkers [38]. The 3D chitosan, *GA*, ML- and HL-3D chitosan-GA were dissolved in acidified water at pH 3.5, obtaining concentrations ranging from 0.05 to 4.0 mg/mL, and analyzed to evaluate the antioxidant activity. In addition, we also measured the antioxidant activity of GA at pH 6.0 and 7.0. Briefly, 200 µL of 0.4 mM DPPH solution in methanol was mixed with 50 µL of each sample in a 96 well microplate. The mixture was shaken vigorously and kept in the dark at room temperature for 30 min. Finally, the absorbance was measured at 517 nm, and the inhibition of DDPH was interpreted by evaluating the colour change in the wells (violet → yellow). The value of DPPH inhibition was calculated as follows:

$$DPPH\;radical\;inhibition\;\left(\%\right)=\frac{A_{control}-A_{sample}}{A_{control}}\times 100$$

where *A_control_* and *A_sample_* are the absorbance of *DPPH* following the addition of either 50 µL of pure water or 50 µL of 3D chitosan, *GA* and 3D chitosan-*GA* solutions, respectively.

### 2.10. Antimicrobial Activity

For this study, we used XL10-Gold (200314 Stratagene) as *E. coli* strain and *S. aureus* – Xen31 (119242 Perkin Elmer, Milano, Italy) as the bioluminescent pathogenic bacteria. All microbial strains were stored at –80 °C in cryovial and 40% (v/v) glycerol. The bacteria were grown overnight at 30 °C in Luria Bertani (LB) medium, from which an inoculum was taken and adjusted to an OD 600 nm of 0.20 ± 0.02 (1 × 10^8^ colony-forming units [CFU]/mL). Subsequently, for each bacterium, at least four wells of sterile 96-well microtiter plates were filled with cells and sample (3D chitosan, GA, and HL-3D chitosan-GA). These were tested at two different concentrations (1.0 and 0.5 mg/mL). Cell suspensions with acidified water and cell suspension without sample were used as controls. The microtiter plates were then incubated for 24 h at 30 °C in an orbital shaker (150 rpm). The absorbance was measured at 600 nm using the EnSpire Multimode Plate Reader (Perkin Elmer, Milano, Italy). 

### 2.11. Statistical Analysis

All data are presented as mean ± standard deviation (n = 4). The SPSS program (rel. 20, IBM, Armonk, NY, USA) was used to analyze the statistical significance (*p* < 0.05), which was determined using the Student’s *t*-test. 

## 3. Results and Discussion

### 3.1. Preparation and Characterization of 3D Chitosan-GA Complexes

The bioactivity of 3D chitosan-GA complexes depends on the number of GA molecules adsorbed on the 3D chitosan structures. Therefore, to find the best experimental conditions that permitted the maximum amount of GA to be adsorbed onto the 3D chitosan matrix, we performed preliminary experiments at various pH (from 3.0 to 9.0) by adding 1 mM GA solution to 100 mg 3D chitosan in a final volume of 10 mL. Control tests on the two individual components were also performed at each pH. The adsorption results demonstrated the maximum adsorption at pH 4.0 (Table S1). A gradual change in the color of the aqueous GA solution after NaOH addition passing from pH 4.0 to pH 9.0 was observed. Indeed, the initially colorless solution changed to orange and then to green as the pH increased. This phenomenon can be explained by the oxidation of GA and the formation of polymerized *o*-quinone derivatives, facilitated by alkanization, resulting in the green coloration of the medium [38]. Moreover, as reported by Toth and collaborators [39], the typical 265 nm absorption characteristic of GA solutions at low pH shifted to 300 nm with increasing pH. On the contrary, the 3D chitosan structures in the solution were always colorless regardless of the pH. However, their solubility was higher at pH < 4.0 and decreased at pH ≥ 4.0, where the formation of an opaque and gelatinous system occurred. For these reasons, all the loading experiments were carried out at pH 4.0, with this pH value being correct compromise that allowed for the fulfillment of two requirements, i.e., the protection of the 3D chitosan structures and the preservation of GA redox state. Once selected pH 4.0 as the optimal adsorption pH using the procedure described in Section 2.4, we determined the amount of GA adsorbed onto 3D chitosan samples after 24 h of incubation with 10 mL of either 1.0 mM GA (low loading), 15.0 mM GA (medium loading), or 23.5 mM GA (high loading) (Table 2).

**Figure 1:**
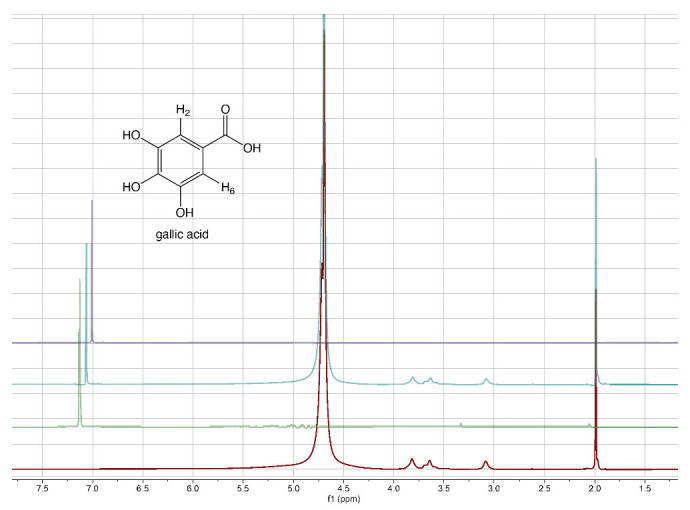
^1^H-NMR spectra of 3D chitosan at pH 3.5 (red), GA at pH 3.5 (green), HL-3D chitosan-GA complex at pH 3.5 (cyan), and GA at pH 6.0 (purple). All spectra were recorded at 25 °C in 9:1 H_2_O/D_2_O.

From literature data [40], it emerged that the grafting efficiency of GA onto chitosan was, at the best studied conditions, equal to 285.9 mg GA/g chitosan, corresponding to 1680 mmol/kg. Other authors [41] reported that the highest grafting degree, expressed as 88.5 mg of GA equivalents (GAE) per g of chitosan-GA (520 mmol/kg), was obtained when the mass ratio of GA to chitosan was 0.5:1. The comparison of the literature data with those reported in Table 2 reveals that the approach proposed here for the preparation of 3D chitosan–GA complexes is more efficient, other than rapid and ecofriendly, than previous methods based on grafting methodologies. To confirm the adsorption of GA into the 3D chitosan structure and assess the entrapped GA’s chemical stability, we recorded the ^1^H-NMR spectra of chitosan and GA before and after their mixing. To maximize the water solubility of chitosan and improve the quality of NMR spectra, we adjusted the pH of all NMR samples from 4.0 to 3.5. The water-suppressed NMR spectra of GA, 3D chitosan, and HL-3D chitosan-GA complex are showed in Figure 1. The proton NMR spectrum of the water-dissolved GA displayed one singlet signal attributable to the homotopic H2 and H6 aromatic protons resonating at 7.13 ppm (Figure 1, green spectrum). In the 3D chitosan-GA complex (Figure 1, cyan spectrum), we observed the GA characteristic aromatic singlet resonating at 7.06 ppm (upfield-shifted by 0.07 ppm). The remaining signals, all belonging to the 3D chitosan structure, were perfectly superimposable to those of the pure 3D chitosan recorded in the same conditions (Figure 1, red spectrum). Thus, the NMR analysis confirmed the successful incorporation of GA into the 3D chitosan structure, as well as the preservation of its redox state. Moreover, we recorded the NMR spectra of pure GA at pH 6.0 to also demonstrate the preservation of the GA structure at the pH that was used for the release kinetics study reported in Section 3.2. The resulting NMR spectrum, reported in purple in Figure 1, displayed the presence of a single singlet attributable to the homotopic H2 and H6 aromatic protons of GA, thus confirming the chemical stability of GA at pH 6.0.

**Figure 2:**
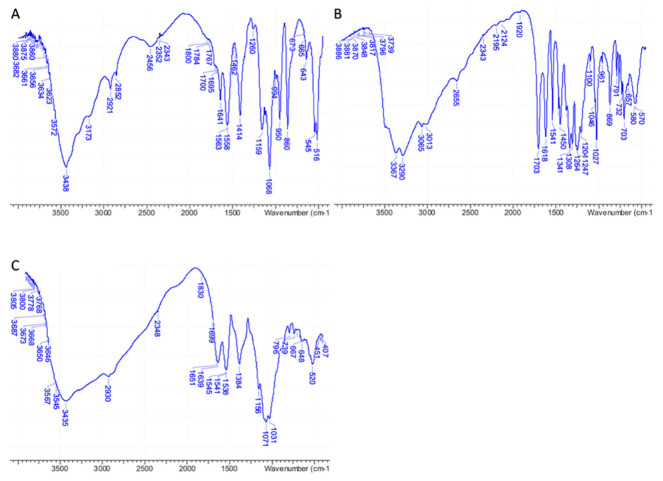
FT-IR spectra of pure 3D chitosan (**A**), GA (**B**), and ML-3D chitosan-GA complex (**C**).

IR analyses performed on pure 3D chitosan, GA, and ML-3D chitosan-GA complex were fully in agreement with the NMR data, confirming the adsorption of GA onto the 3D chitosan matrix. A wide band corresponding to the stretching vibrations of 3D chitosan’s O–H and N–H was observed between 3000 and 3600 cm^–1^ in the IR spectrum of pure 3D chitosan (Figure 2A). The IR bands of pure GA’s phenolic hydroxyls were observed as an even wider band between 2600 and 3600 cm^–1^ (Figure 2B). As a result of the contribution of the two components, the high wavenumber region of the IR spectrum of the 3D chitosan–GA complex showed a wide band covering a 2600–3600 cm^–1^ range (Figure 2C). A further diagnostic was the change observed for the chitosan’s amide II band that moved from 1558 cm^–1^ (Figure 2A) to 1538 cm^–1^ after the adsorption of GA onto the 3D chitosan matrix (Figure 2C) [42].

Table 3 shows the effect of GA adsorption on the porosity of 3D chitosan structures. In this study, the porous scaffolds were fabricated by a lyophilization method, using a vacuum-pump system to sublime ice crystals from polymer matrix, according to the method described in Section 2.2. Not significant differences in porosity were observed for the different 3D chitosan-GA preparations (LL, ML, and HL). Accordingly, the porosity value reported in Table 3 represents the mean value of all preparations. The 3D chitosan structures demonstrated a porosity higher than 90%, whilst GA adsorption following incubation with GA slightly decreased porosity values to about 82%. 

The porosity values are in agreement with the previously reported experiments [35,43]. Porosity values can also be expected to significantly affect structure-degradation kinetics and mechanical properties. The proposed methodology could be adopted as a straightforward and reproducible way to obtain 3D porous structures, by adopting specific parameters (i.e., controlled freezing rate). 

**Figure 3:**
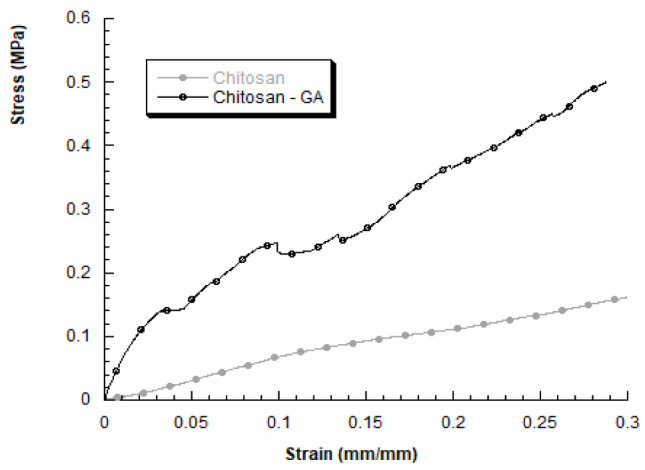
Stress–strain curves obtained for 3D chitosan before and after incubation with 23.5 mM GA to obtain the HL-3D chitosan-GA complex.

We also evaluated the mechanical properties of 3D chitosan structures before and after GA adsorption, in dry conditions. A typical stress–strain curve obtained for 3D chitosan structures is reported in Figure 3 (grey curve). The compressive modulus resulted higher for HL-3D chitosan-GA complex, than the neat chitosan structure. In particular, the obtained results for HL-3D chitosan-GA complex highlighted a stress–strain curve characterized by an initial linear region followed by a change in the slope, indicating mechanical weakening and progressive structure failures, until a maximum stress value was reached (Figure 3, black curve). The HL-3D chitosan-GA complex did not exhibit a typical behavior of flexible foams [44] or 3D scaffolds manufactured through fused-deposition modeling [45]. Additionally, Table 4 reports the maximum stress values measured at a strain level of 0.3 mm/mm and the compressive modulus evaluated as the slope of the initial linear region of the stress–strain curve.

The adsorption of GA via the proposed methodology may lead to an improvement in the compressive modulus and the maximum stress (at 0.3 mm/mm). Anyway, the obtained results agree with previous works performed by other researchers, highlighting the dependence between the internal morphology and the mechanical behavior of freeze-dried chitosan samples [46].

### 3.2. Release of GA from 3D Chitosan-GA Complexes

**Figure 4:**
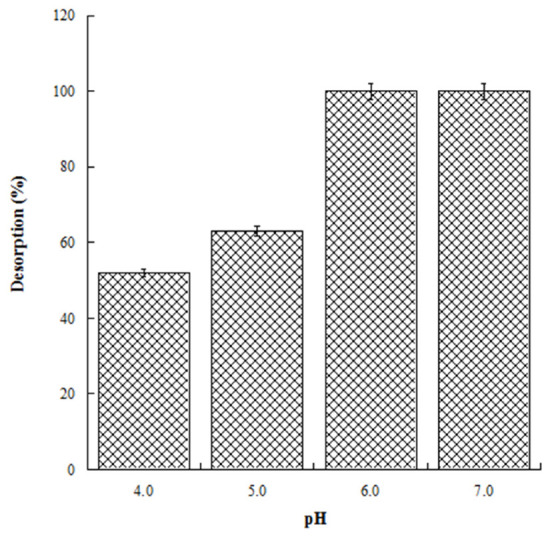
Release of GA from HL-3D chitosan-GA complexes at pH 4.0, 5.0, 6.0 and 7.0 after 24 h of incubation time.

**Figure 5:**
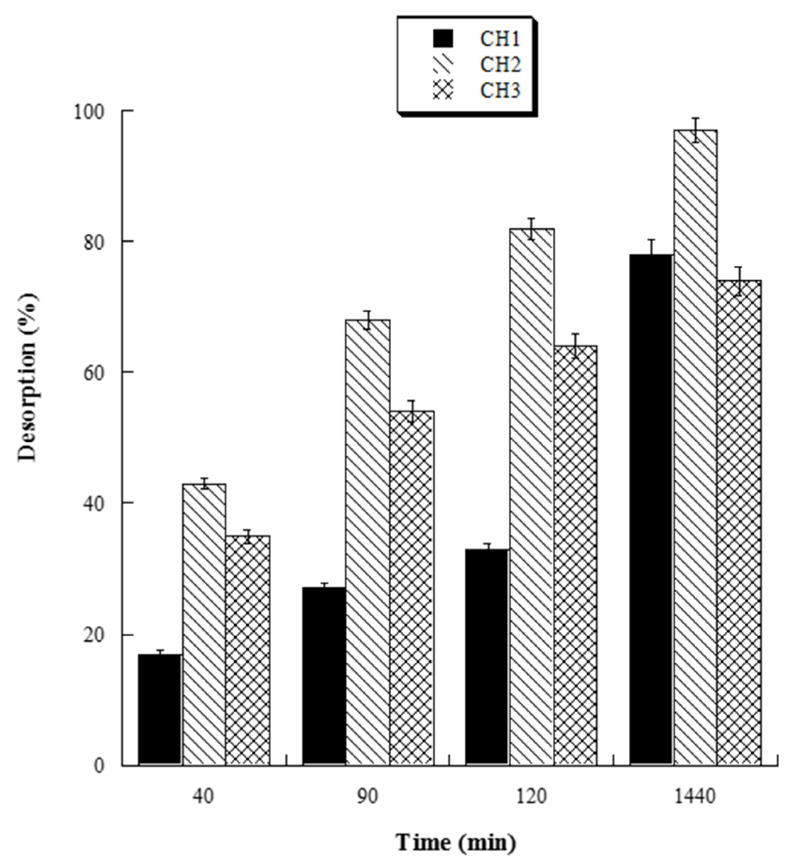
Percentage amount of cumulative GA released from dried HL-3D chitosan-GA at any time point.

With the aim to evaluate the best conditions for GA release, we carried out several experiments at different pH values (4.0, 5.0, 6.0, and 7.0). Four HL-3D chitosan-GA samples were prepared at pH 4.0 (the best loading pH value) and then treated with ultrapure water at different pH values as reported above. The concentration of GA desorbed after 24 h incubation from each 3D chitosan complex was determined by a UV analysis at 265 nm. The results, summarized in Figure 4, revealed that a pH ≥ 6.0 was required for the complete release of the adsorbed GA. Considering that pH 6.0 was determined as the best pH value for both the antioxidant activity and release of GA, we decided to perform the kinetic release study of GA from the 3D chitosan matrix at pH 6.0. To assess the influence of drying time on the release kinetics, three samples of HL-3D chitosan-GA were dried for 24 h (CH1), seven days (CH2), and 14 days (CH3) before assessing the amount of GA released at different time points (40, 90, 120, and 1440 min) following the addition of 10 mL of ultrapure water at pH 6.0. For CH1 sample, we observed an incomplete and nonlinear release of GA (Figure 5, black bars) characterized by a slow release in the first 120 min (33%) followed by a strong acceleration in the succeeding 22 h (78% release after 24 h). In the case of CH3, we observed an almost linear release kinetics along the monitored 24 h (Figure 5, squared bar). However, also in this latter case the percentage of GA released after 24 h was limited to 74%, probably due to the formation of crosslinking phenomena between 3D chitosan and GA. Finally, the CH2 sample, dried for seven days, displayed the best release kinetics, characterized by a linear behavior and an almost complete release at 24 h (Figure 5, diamond bar). Overall, the release kinetics study indicated that a drying time of seven days is the optimal compromise to achieve the complete and linear release of GA from the 3D chitosan matrix.

### 3.3. Antioxidant Activity of 3D Chitosan-GA Complex

**Figure 6:**
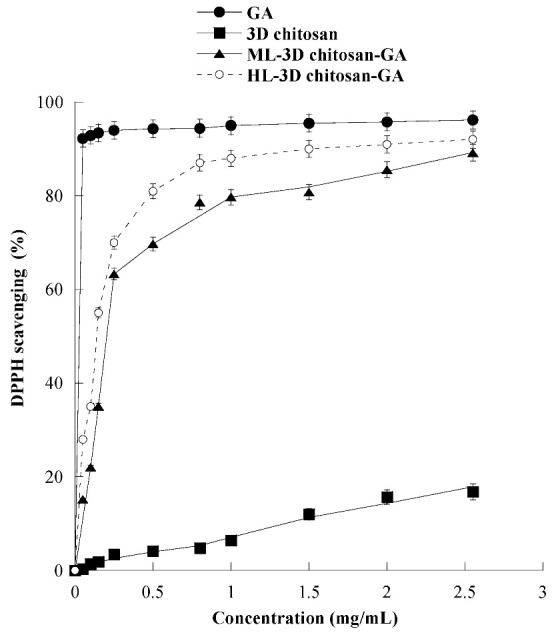
Scavenging activity against the DPPH radical of 3D chitosan, GA, ML-3D chitosan-GA, and HL-3D chitosan-GA at pH 3.5. The GA equivalent in ML- and HL-3D chitosan complex was 168.42 and 342.79 g kg^−1^, respectively. 3D-chitosan-GA samples were dried for seven days before measurements.

The antioxidant activity of 3D chitosan, GA, ML-3D chitosan-*GA*, and HL-3D chitosan–GA was determined by using the DPPH radical scavenging assay. In this assay, an antioxidant molecule reduces the DPPH radical (a deep purple compound) to diphenylpicrylhydrazine (a yellow/colorless compound), and the extent of discoloration depends on the hydrogen-donating ability of the antioxidant molecule. The results, shown in Figure 6, revealed that by increasing the 3D chitosan concentration from 0.05 to 2.55 mg/mL its scavenging activity raised up to 16.8% inhibition, in agreement with the literature data [47]. This modest inhibitory activity may be partly due to the occurrence of inter- and intra-molecular hydrogen links within the chitosan matrix [47]. On the contrary, as expected, GA showed an excellent DPPH radical-scavenging activity (92.3%), also at the lowest tested concentration (0.05 mg/mL). This activity was dramatically higher than that of 3D chitosan (0.4%), ML-3D chitosan-GA (15.2%), and HL-3D chitosan-*GA* (28.0%). The scavenging activity of the 3D chitosan-GA complexes increased significantly at higher concentrations, reaching 90% DPPH inhibition at 2.55 mg/mL. This value was only slightly lower than that observed for the free GA molecule (93%) at the same concentration and is significantly higher than the 80% value reported for chitosan-GA complexes obtained by GA grafting via carbodiimide coupling [28,48]. The data shown in Figure 6 refer to the experiments conducted using ML- and HL-3D chitosan–GA preparations which were dried for seven days before measurements.

**Figure 7:**
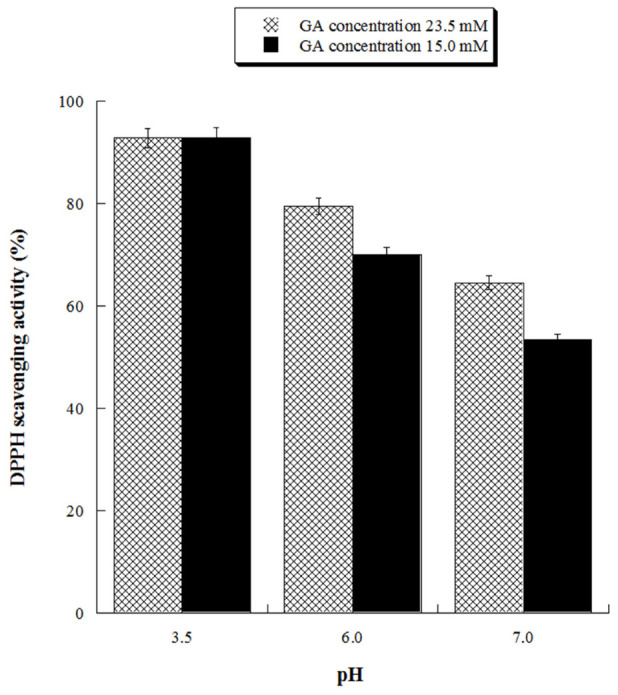
Scavenging activity against DPPH radicals of gallic acid (GA) at pH 3.5, 6.0 and 7.0. GA 23.5 mM concentration is equivalent to 4.0 g/L, GA 15 mM concentration is equivalent to 2.55 g/L.

With the aim of assessing the antioxidant activity of GA at ecofriendly working conditions, we evaluated its antioxidant activity at neutral (pH 7.0) and close neutral pH (pH 6.0) for two different concentrations (15.0 and 23.5 mM). The results, reported in Figure 7, demonstrated that GA solutions possess significant antioxidant activity also at pH 6.0, reaching 80% DPPH inhibition at the highest tested GA concentration. 

### 3.4. Antibacterial Activity 

**Figure 8:**
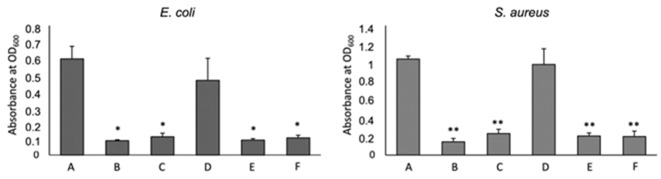
Effect of Chitosan (CS) and Gallic Acid (GA) on bacterial growth. A: non treated, B: HL-3D chitosan-GA 1 mg/mL, C: HL-3D chitosan-GA 0.5 mg/mL, D: CS 10 mg/mL, E: GA 1 mg/mL, F: GA 0.5 mg/mL. The mean ± SD for at least four replicates is shown. Statistical significances between treated bacteria versus untreated * *p* < 0.001, ** *p* < 0.0001.

To test the antimicrobial capacity of 3D chitosan-GA complexes against Gram-negative and Gram-positive bacteria, seven days-dried HL-3D chitosan-GA complexes were mixed with *E. coli* (G–) and *S. aureus* (G+) cultures that were selected as the model strains. The effect of two different concentrations (0.5 and 1.0 mg/mL) of HL-3D chitosan-GA against the two bacterial strains is displayed in Figure 8 in comparison with that of 3D chitosan and GA alone. 

GA, rather than the chitosan, inhibited the bacteria growth both at 1.0 and 0.5 mg/mL. 3D chitosan–GA complexes demonstrated significantly higher antimicrobial capacity, with an inhibition percentage of around 83% (*p* < 0.001 for *E. coli*, *p* < 0.0001 for *S. aureus*) (Figure 8, bars B and C), than 3D chitosan alone (Figure 8, bars D), regardless of the bacterial strain and concentration used, indicating that the adsorption of GA effectively increases the antimicrobial activity of 3D chitosan. Borges et al. (2013) [49] reported that ferulic acid and gallic acid led to irreversible changes in membrane properties (charge, intra and extracellular permeability, and physicochemical properties) through hydrophobicity changes, decrease in negative surface charge, and occurrence of local rupture or pore formation in the cell membranes with consequent leakage of essential intracellular constituents.

## 4. Conclusions

In the present study, we have reported the preparation of non-covalent 3D chitosan–GA complexes using a simple, sustainable, and eco-friendly adsorption procedure. The successful incorporation of GA into the 3D chitosan structure was confirmed by FTIR, NMR, and compression tests. Loading and releasing studies performed at various pH indicated 4.0 and 6.0 as the optimal loading and releasing pH, respectively. Up to 2015 mmol GA/kg were incorporated when 3D chitosan was incubated with 23.5 mM GA at pH 4.0. More importantly, we confirmed that the 3D chitosan-adsorbed GA retains its antioxidant and antimicrobial activities, as demonstrated by DPPH and inhibitory assays on selected Gram + and Gram – bacteria strains. Considering the benefits offered by the incorporation of actives into chitosan matrixes (i.e., chemical protection and prolonged release), we believe that the approach proposed here may find proficuous application in the food industry, as well as in the nutraceutical, pharmaceutical and cosmeceutical fields.

## Figures and Tables

**Table 1:** 3D chitosan-GA complexes obtained by contacting 100 mg 3D chitosan samples with three different concentrations of GA.

Gallic acid Concentration (mM)	3D Chitosan-GA Complexes with Different GA Loadings
1.0	Low Loading, LL-3D chitosan-GA
15.0	Medium Loading, ML-3D chitosan-GA
23.5	High Loading, HL-3D chitosan-GA

**Table 2:** Adsorption of GA on 3D chitosan structures at pH 4.0 for an incubation time of 24 h.

GA Concentration (mM)	GA Adsorbed on 3D Chitosan Structures (mmol/kg)
1.0	111 ± 3.66
15.0	990 ± 3.90
23.5	2015 ± 4.64

Values are given as mean ± Standard Deviation (SD) (n = 4).

**Table 3:** Results from porosity measurements of 3D chitosan before and after GA adsorption.

3D Structure	Porosity (%)
3D chitosan	94.91 ± 0.36
HL-3D chitosan-GA	82.13 ± 1.09

**Table 4:** Compressive modulus *E* and maximum stress (σ_max_) at a strain level of 0.3 mm/mm for 3D chitosan and HL-3D chitosan-GA complex.

Sample	Compressive Modulus E (MPa)	Maximum Stress σ_max_ (MPa)
3D chitosan	0.7 ± 0.1	0.12 ± 0.02
HL-3D chitosan-GA	6.0 ± 1.2	0.51 ± 0.13

## Supplementary Materials

The following are available online at https://www.mdpi.com/article/10.3390/gels8020124/s1, Table S1: Amount of GA adsorbed on 3D chitosan at different pH values after incubation with 1 mM GA for 24 h.
