# Supplementary material for: 3D Chitosan-Gallic Acid Complexes: Assessment of the Chemical and Biological Properties
Source: Gels. 2022 Feb 15;8(2):124. doi: 10.3390/gels8020124 (PMC8872007; doi:10.3390/gels8020124)
Supplement: Supplementary file 1 [file gels-08-00124-s001.zip › gels-1585072-supplementary.pdf]

gels

MDPI

Article

3D Chitosan-Gallic Acid Complexes: Assessment of the Chemical and Biological Properties

Maria Marzano[1], Nicola Borbone[2,3], Felice Amato[4,5], Giorgia Oliviero[3,4], Pierpaolo Fucile[6], Teresa Russo[7], Filomena Sannino[8,*]

[1] Institute of Applied Sciences and Intelligent Systems Unit of Naples, National Research Council, Via Pietro Castellino 111, 80131 Naples, Italy; maria.marzano@na.isasi.cnr.it
[2] Department of Pharmacy, University of Naples Federico II, Via Domenico Montesano 49, 80131 Naples, Italy; nicola.borbone@unina.it
[3] ISBE-IT, University of Naples Federico II, 80138 Naples, Italy
[4] Department of Molecular Medicine and Medical Biotechnologies, University of Naples Federico II, Via Sergio Pansini 5, 80131 Naples, Italy; felice.amato@unina.it, giorgia.oliviero@unina.it
[5] CEINGE-Biotecnologie avanzate, Via Gaetano Salvatore 486, 80131 Naples, Italy
[6] Department of Advanced Biomedical Sciences, University of Naples Federico II, Via Sergio Pansini 5, 80131 Naples, Italy; pierpaolo.fucile@unina.it
[7] Institute of Polymers, Composites and Biomaterials, National Research Council, Viale J. F. Kennedy 54, Pad. 20 Mostra d'Oltremare, 80125 Naples, Italy; teresa.russo@cnr.it
[8] Department of Agricultural Sciences, University of Naples Federico II, Via Università 100, 80055 Portici, Naples, Italy; fsannino@unina.it
* Correspondence: fsannino@unina.it

Table S1. Amount of GA adsorbed on 3D chitosan at different pH values after incubation with 1 mM GA for 24 h.

pH	GA Adsorbed (mmol/kg)
3.0	41 ± 1.53
4.0	111 ± 3.66
5.0	109± 3.55
6.0	109± 3.43
7.0	108± 3.20
8.0	105± 3.22
9.0	100± 3.00
